# Positive Health beyond boundaries in community care: design of a prospective study on the effects and implementation of an integrated community approach

**DOI:** 10.1186/s12889-019-6551-5

**Published:** 2019-02-28

**Authors:** Sanneke J. M. Grootjans, M. M. N. Stijnen, M. E. A. L. Kroese, A. J. M. Vermeer, D. Ruwaard, M. W. J. Jansen

**Affiliations:** 1 Department of Health Services Research, Care and Public Health Research Institute (CAPHRI), Faculty of Health, Medicine and Life Sciences, Maastricht University, Duboisdomein 30, 6229 GT Maastricht, The Netherlands; 2 Public Health Service South Limburg (GGD Zuid Limburg), Het Overloon 2, 6411 TE Heerlen, The Netherlands

**Keywords:** Integrated community approach, Quadruple aim, Positive health, Bottom – Up approach, Quasi – Experimental design

## Abstract

**Background:**

High healthcare expenditures due to population ageing and chronic complex health complaints are a challenge on a global scale. To improve the quality of healthcare, population health, and professionals’ work satisfaction and to reduce healthcare costs (Quadruple Aim), the Dutch Ministry of Health, Welfare and Sport designated nine pioneer site regions across the Netherlands. One of these pioneer sites is the integrated community approach (ICA) known as ‘Blue Care’. This article describes the design of a prospective study investigating the effects of Blue Care ICA on Quadruple Aim outcomes and a process evaluation focussing on its implementation in deprived neighbourhoods.

**Methods:**

A mixed-methods approach, combining both quantitative and qualitative research methods, is applied to yield an enriched understanding of the various processes that will take place in the neighbourhoods.

A prospective, quasi-experimental study is conducted within a natural experiment. Blue Care ICA is being implemented between 2017 and 2020 and research activities are taking place parallel to the implementation process. Effects of Blue Care ICA are measured at T0 (baseline), T1 (after 1 year), T2 (after 2 years) and at T3 (after 3 years) using a questionnaire. The primary outcome measure is health-related quality of life (SF-12v2), secondary outcomes are health status (EQ-5D-5 L), resilience (RS-Scale), Positive Health (Spiderweb diagram) and quality of care (grade 0–10). As part of the process evaluation, the Consolidated Framework for Implementation Research guided the formulation of process evaluation questions. Participant observations, interviews and focus groups with all stakeholders active in the Blue Care ICA will be conducted during the whole implementation period (2017–2020).

**Discussion:**

The evaluation takes into account the interconnections between content, application, context and outcomes to understand how the Blue Care ICA unfolds over time in a complex, dynamic setting. Results of the effect and process evaluation will become available in 2020.

**Trial registration:**

NTR 6543, registration date; 25 July 2017.

## Background

In 2015 the United Nations’ [1] (UN) sustainable development agenda was launched containing seventeen sustainable development goals (SDG) aiming to promote prosperity and well- being for all over the next fifteen years. Although all goals can be seen as interrelated to each other, one goal is of particular importance here, namely ‘ensuring healthy lives and promote well-being for all at all ages’ [2]. In realizing the SDG agenda, an integrated approach is considered of utmost importance. Integrated health care (IHC) has also become an important focus in the health care sector on a global scale, since the world is confronted with a disease burden shift from communicable diseases to non-communicable diseases [3]. In addition, population ageing and thereby an increase in the number of people who suffer from complex and/or multiple (chronic) health complaints is steadily increasing the burden on health care expenditures worldwide [4]. As a response to this, Berwick and colleagues [5] formulated the Triple Aim goals to improve health system performance by improving the health of populations, enhancing the patient experience of care and reducing per capita cost of health care. In addition Bodenheimer and colleagues [6] proposed one more dimension to expand the Triple Aim goals to the Quadruple Aim goals, by adding the goal of improving the work life of health care providers, including clinicians and staff.

**Fig. 1:**
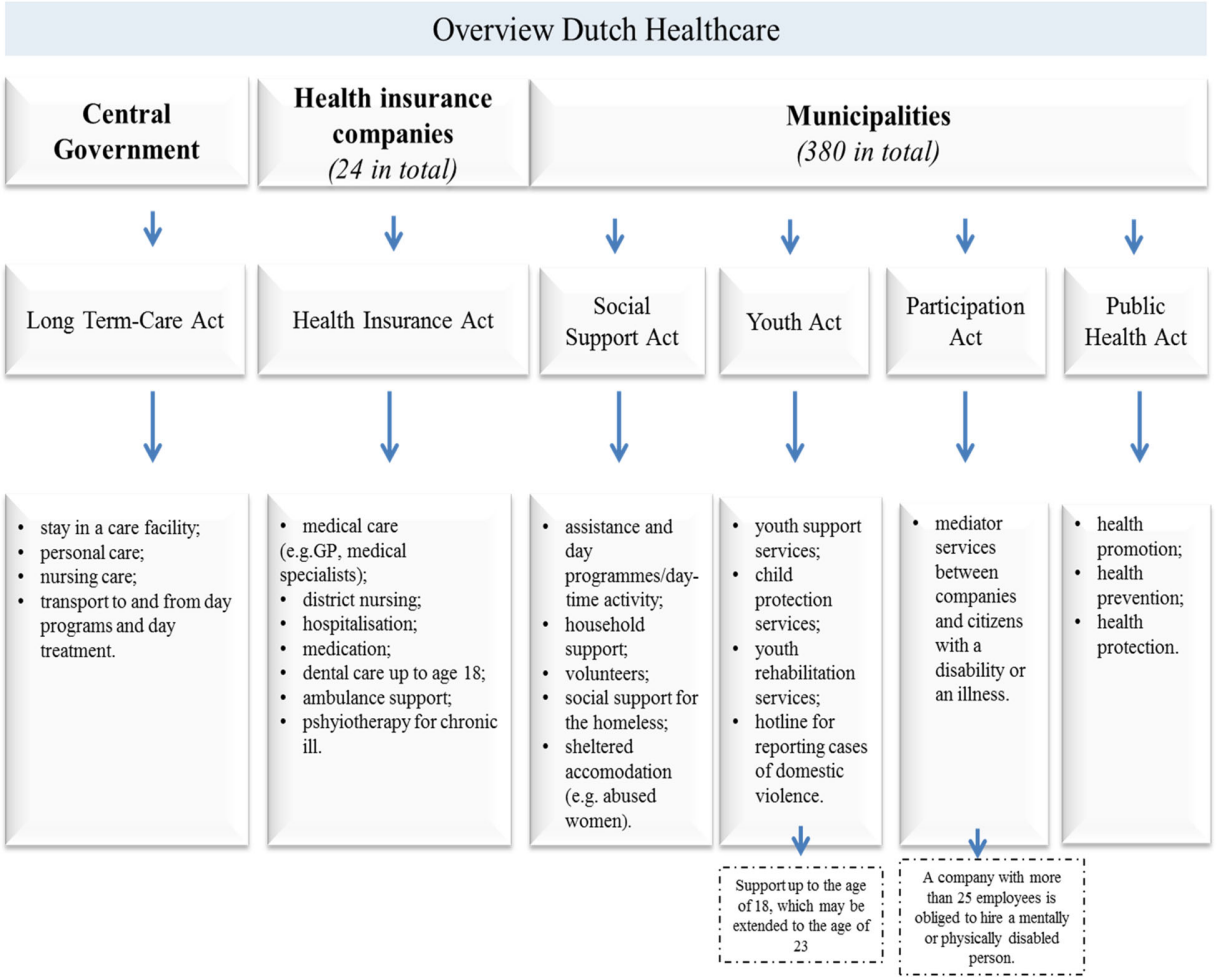
Overview Dutch Healthcare

In the Netherlands, the average amount of money spent on health care per person was considerably higher than the OECD average [7]. These developments seriously threaten future accessibility, affordability and, hence, sustainability of Dutch health care [8]. Health and social care in the Netherlands is the shared responsibility of both the central government and the municipalities. Four basic healthcare related acts form the foundation of the Dutch healthcare system: the Health Insurance Act (Zorgverzekeringswet), the Long Term-Care Act (Wet langdurige zorg), the Social Support Act (Wet maatschappelijke ondersteuning) and the Youth Act (Jeugdwet) [8, 9]. In addition (1) the Participation Act (Participatiewet) supports mentally or physically challenged citizens’ participation in society and (2) the Public Health Act (Wet Publieke Gezondheid) is responsible for public health, prevention, health promotion and health protection by the municipalities at a local authority level. Every 4 years municipalities have to formulate a public health strategy for their area. Figure 1 shows an overview of the basic health and social care Acts and their responsibilities [8–10].

In order to understand the current Health and Social care system a few recent changes have to be explained.

First, in 2006, the new Health Insurance Act entered into force and transformed the Dutch healthcare system to a demand-driven system from a supply-driven system. As a consequence, every citizen living or working in the Netherlands is obliged to have a basic statutory healthcare insurance, purchased at a private insurance company, which covers basic healthcare [10]. Additionally, supplementary private (voluntary) health insurance differs per person and depends on the needs and wishes of the person. There are a total of 24 healthcare insurers active in the Netherlands. Although the healthcare system in the Netherlands can be considered as a semi-free market system, the government plays, among others, a controlling role.

Second, up to 2015 social health care was mainly the responsibility of the central government. Since 2015, health and social care in the Netherlands has transitioned from a centralized system to a decentralized system. The motivation for this transition is to promote an integrated approach tailored to people’s needs and their living condition at the local or neighbourhood level and to keep healthcare affordable. Moreover, the transition aims to encourage people to draw on their own network, resilience and resources for support. As a consequence of the transition in 2015, all 380 municipalities in the Netherlands have their own responsibility over the social domain (Social Support Act, the Youth Act and the Participation Act) and develop their own policies, based on local population needs.

The recent transition of 2015, in the health and social care domain poses challenges to local authorities in terms of sustainability of care, particularly in the South Limburg region in the south of the Netherlands. Population ageing is especially pronounced there and the population suffers from decreased health and a lower life expectancy compared to the province of Limburg and the Netherlands as a whole [11]. Among others, complex care consumers often need care financed under both the healthcare as well as the social care domain, which is argued to be challenging due to fragmentation of care and silo thinking of professionals involved. Moreover, fragmentation in health and social care systems is one of the reasons worldwide that health and social care cannot live up to the needs of the patients [12]. Hence, worldwide, in western countries, there is a focus on diminishing the gap between health care systems and social care services [13]. For example the Accountable Health Community (AHC) model in the United States, which is implemented by the Centers for Medicare and Medicaid Services (CMS), aims to connect healthcare to social services, as a result reducing emergency department visits by 9% [14]. Similarly, the U.K. aims to fully offer effective integrated health and social care services to their population by 2020 to meet the rising demands of the population [14, 15]. By integrating both the health and the social care domain the patient is staged in the center again, instead of positioned in between different services and organizations.

In order to create a sustainable healthcare system in the Netherlands, the Ministry of Health, Welfare and Sport selected nine innovative regions as pioneer sites to experiment with population management [16] to achieve the Quadruple Aim goals. Blue Care in the Maastricht-Heuvelland region in the southern part of Limburg province is one of the nine pioneer sites appointed by the Ministry of Health, Welfare and Sport. The term Blue Care represents sustainable care, since the term ‘Blue’ is inspired by the frequently used color for sustainability ‘Green’.

At the pioneer site Blue Care, health care organizations, patient organizations and the health care insurers’ providers have committed themselves to achieving the Quadruple Aim goals and pledged that they will prioritize population health above their organizational goals.

### The integrated community approach blue care

One of the initiatives developed as part of the Blue Care pioneer site is the Blue Care integrated community approach (ICA), implemented in four low socio-economic status neighbourhoods in Maastricht, a city in the south of the Netherlands. Before the starting phase of the Blue Care ICA in 2016, stories and cases were collected based on conversations with citizens and health and social care professionals in the four neighbourhoods to gain an understanding of the current facilitators and barriers regarding the health and social care domain in the four communities.

As a result of these findings, sub-projects are initiated in the Blue Care ICA in which suggested improvements are being put into practice on a small scale while using a bottom-up approach. These various sub-projects need to fulfill the following criteria: the project needs to cover both the health and the social care domain; the project needs to correspond with the needs of citizens and professionals; and the project needs to be sustainable over time (in terms of continuation after the implementation period). Furthermore, projects are developed ‘on the go’, meaning that feedback is gathered during the implementation process and improvements and adjustments are made for the project to fit to citizen’s and professional’s needs.

The Blue Care ICA is implemented in four neighbourhoods with low socioeconomic status (SES): Limmel, Nazareth, Wittevrouwenveld and Wyckerpoort [17]. Low socioeconomic status is often linked to decreased health and lower life expectancy, which often affects, among other things, the ability of people to participate in society [18]. Also, low socioeconomic status communities appears to have lower social cohesion [19], which is a possible mediator between neighbourhood deprivation and health [20]. For example, less socially cohesive neighbourhoods are associated with increased depression and lifestyle problems (i.e., smoking, lack of exercise) [21]. Furthermore, poverty and unemployment are significantly associated with the duration of episodes of common mental disorders such as anxiety and depression. Therefore, the need to stimulate participation and to address the health and well-being of citizens is especially important in low socioeconomic status communities to decrease socioeconomic health inequalities and inequities [22] and to decrease costs of care [23, 24]_._

### Positive health as a shared ideology

**Fig. 2:**
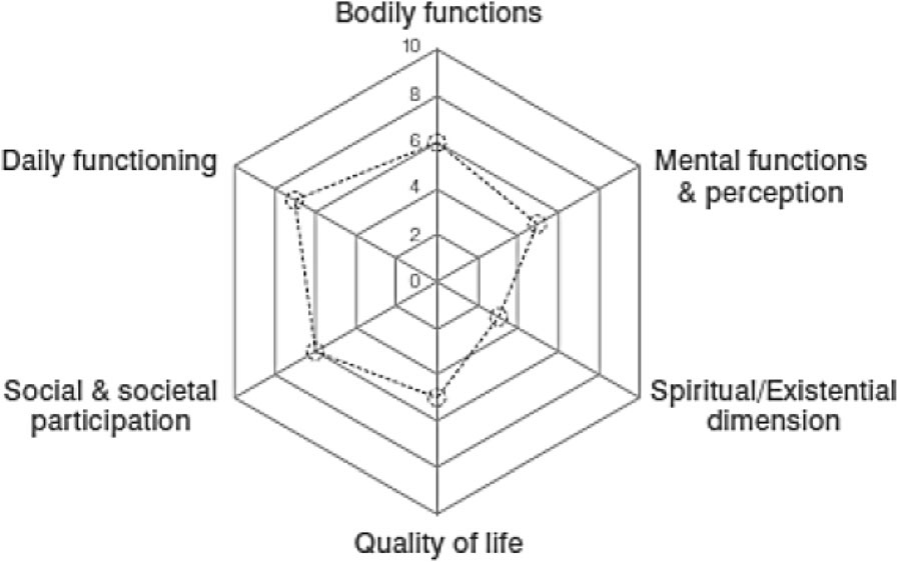
The six dimensions of Positive Health

The aim of Blue Care ICA is to improve population health and the perceived quality of life by implementing a community approach, based on the ideology of Positive Health. Positive Health is based on a new vision on health, where health is described as ‘the ability to adapt and self – manage’ [25]. The new description of health was introduced in 2011, since the traditional World Health Organization (WHO) definition of health as ‘a state of complete physical, mental and social well-being and not merely the absence of disease or infirmity’ was no longer suitable, with the rising numbers of non-communicable diseases. Additionally, according to the traditional WHO description of health, almost everybody can be considered ill to some extent, since the description uses a static state of health. The new description of health considers health from an asset-based perspective that goes beyond focusing of disease/illness by also including the individual’s perceived sense of control and coping with life events [25, 26]. Based on this line of reasoning, Positive Health aims to enhance citizens’ strengths and self-reliance and consists of six dimensions (Fig. 2), which together encompasses a holistic view of health [27]. In the Blue Care ICA approach, sharing and implementing the ideology of Positive Health among citizens, professionals and policymakers may contribute to decreasing fragmentation in health, since Positive Health combines both aspects of health and social care, and creates a collective language between the professionals working in the different domains.

### Three core elements

Positive Health is used as an overarching ideology that runs through the veins of Blue Care. Apart from that, three core elements are designated that encompass the building blocks of Blue Care.

#### Citizens in action

The first core element aims to change citizens’ attitude, self-efficacy and behavior to give meaning to their own lives and stimulate societal participation (‘citizens in action’). Citizens are actively involved in Blue Care and are stimulated to address bottlenecks and develop projects which address these bottlenecks. Recent findings in the intervention neighbourhoods from the perspective of the citizens showed that there is a lack of communication between health and social care professionals, there is too much bureaucratic delay, and rules and regulations are considered to be inflexible with insufficient attention to the needs of citizens. In addition, citizens are consulted on a regular base during Blue Care by a citizen panel which comes together two times a year facilitated by the researcher and a member of the Blue Care coordination team. Hence, these meetings serve both as a research and a practical implication tool to diminish the burden on the citizens.

#### Professionals in action

The second core element aims to enable and support the professional freedom of health and social care providers to organize (preventive) support in the main interest of the individual citizen, above and beyond organizational interests or financial reason (‘professionals in action’). One of the important bottlenecks mentioned by the professionals during the starting phase of the Blue Care ICA in 2016 was that there is a lack of communication between the different health and social care providers. Contradictions in treatment and advice given between professionals treating the same patient are more the rule than the exception, which is frustrating for professionals involved. Furthermore professionals mentioned ‘being tied up by inflexible rules, regulations and bureaucracy created by the municipality and health care insurer’ as an enormous bottleneck in treating patients efficiently.

#### Combining budgets

The third core element, ‘combining budgets’, will build a reimbursement system in which the budgets for health and social care are combined on a population level. Therefore, the budgets of the dominant health insurer in the south of Limburg (VGZ, Health Insurance Act) will be combined with the budgets of the social care domain (Social Support Act, Youth Act and Participation Act).

The financial reimbursement system is agreed upon at the macro (policy) level and is considered to be a prerequisite for enabling and facilitating the implementation of initiatives at the micro and meso (community and organizational) level. The national budgets of the Long –Term Care Act will not be included in the budget of the Blue Care ICA, even though the costs in this area will be monitored along the way. The objective is that the costs of health and social care, following the implementation of projects within the Blue Care ICA, will be in accordance with available financial budgets (thereby breaking the trend of increasing costs) or, ideally, that projects will lead to a decrease in costs. To judge this, the reimbursements of 2015 in these domains will be used as an upper limit.

### Hypothesis and research questions

The Blue Care integrated community approach aims to improve the health-related quality of life of citizens living in four low socioeconomic status neighbourhoods of Maastricht and thereby reduce socioeconomic health inequalities. We hypothesize that the Blue Care ICA, using a bottom-up approach and Positive Health as the shared ideology, will lead to an improvement of the Quadruple Aim goals.

The research questions are:

1. What are the outcomes of the Blue Care ICA in terms of:
  - effects on the health-related quality of life (18+ years);
  - effects on the perceived quality of care of the citizens;
  - changes in the work satisfaction of professionals working in the four neighbourhoods;
  - changes in total reimbursements at a (sub)population level in the four neighbourhoods and substitution in different types of reimbursements?
2. How is the Blue care ICA embedded in the four neighbourhoods in terms of:
  - inter-professional and inter-organizational collaboration;
  - application of Positive Health and integration into working routines of professionals;
  - delivering person-centred care and support from a generalist perspective;
  - implementation of a combined budget (bundling of budgets in the health and social care domain)?

## Methods/ study design

### Study design

Overall, a mixed-methods approach, combining both quantitative and qualitative research methods, is applied to yield an enriched understanding of the various processes that will take place in the neighbourhoods aiming to improve population health, the perceived quality of care, work satisfaction of professionals and to reduce per capita costs. The Blue Care ICA is being implemented in the four intervention neighbourhoods between 2017 and 2020, and research activities will be conducted parallel to the implementation process. The research focusses on two parts: (1) the effects of the Blue Care ICA on Quadruple Aim outcomes and (2) the implementation process of the Blue Care ICA in the four neighbourhoods of Maastricht.

Our investigation of the effectiveness on population health and (experienced) quality of care of the program utilizes a prospective, quasi-experimental design. The study can best be considered a ‘natural experiment’ [28], as the research will follow the natural course of the development of the Blue Care ICA in daily practice. It aims to test whether it is likely to be effective in routine practice by comparing it to neighbourhoods where this approach is not implemented (comparison neighbourhoods).

**Fig. 3:**
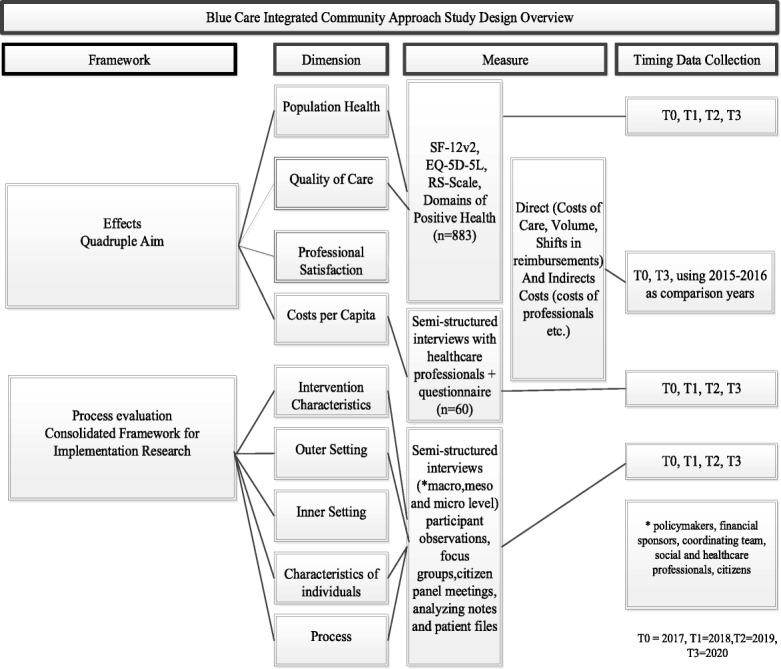
Study Overview

To measure its effects on work satisfaction of professionals and per capita costs, both qualitative and quantitative methods will be used. To determine how the Blue Care ICA is embedded in the four neighbourhoods, a process evaluation plan is designed using the Consolidated Framework for Implementation Research (CFIR) as an overarching framework [29]. An overview of the study design including a global overview of data collection measures and timing of data collection is provided in Fig. 3.

### Target population

The target population of the Blue Care ICA is all citizens who reside in the participating neighbourhoods. Additional inclusion criteria for the study are that citizens should have resided in the neighbourhoods for at least 3 years and should be at least 18 years of age.

The intervention group comprises the four neighbourhoods in which the Blue Care ICA is implemented. A comparison group was selected based on similarity of the ‘comparison’ citizens with their ‘intervention’ counterparts in terms of demographic neighbourhood characteristics (socioeconomic status, ethnicity, use of health and social care domain).

In the intervention neighbourhood, two general practices are located and both agreed to participate in this study.

Of the five general practices located in the comparison neighbourhoods, two general practices agreed to participate in this study. Reasons for non-participation were: not enough time for potential extra tasks and fear of burdening their patients with possibly unwanted questionnaires. All general practices (comparison and intervention) are group practices.

Citizens living in the intervention and comparison neighbourhoods were selected from the Electronic Patient System (EPD) of the participating general practices located in these neighbourhoods. Citizens eligible for participation in the study approached by means of a information letter and consent form, accompanied by a letter from their general practice. Signed consent forms are obtained from all citizens willing to participate in the study.

Table 1 shows the demographic characteristics of the intervention and comparison neighbourhoods [17, 30–32]_._ The age distribution in the intervention neighbourhoods is equal to the average of the comparison neighbourhoods, except for a somewhat higher proportion of those under age 25 (mostly student population, 18–24 yrs) and a lower percentage of people over 65 years in the intervention neighbourhoods. The inhabitants of the intervention neighbourhoods feel slightly more in control over their life compared to the comparison neighbourhoods.

**Table 1:** Demographic characteristics of intervention and comparison neighbourhoods

	Number of Inhabitants *(CBS,2016)*	Age distribution *(CBS, 2016)*	Nonwestern immigrants *(CBS, 2016)*	Inhabitants with a low income^a^ *(Municipality Monitor, 2016)*	Inhabitants with an academic degree (university /university of applied sciences) *(PHS Monitor, 2017)*	Does not feel in control over own life *(PHS, 2017)*	Perceives own health as poor *(PHS, 2017)*	Increased risk of anxiety/Depression *(PHS,2017)*	Experiences severe loneliness *(PHS,2017)*	Is overweight (BMI between 25 and 30)^b^ *(PHS,2017)*
Intervention group										
Nazareth	3.280	0–24 yrs.: 29%	14%	54%	20%	24%	28%	69%	19%	48%
		25–64 yrs.: 55%								
		≥65 yrs. 16%								
Limmel	2.370	0–24 yrs. = 44%	21%	52%	17%	13%	28%	54%	22%	47%
		25–64 yrs. = 44%								
		≥65 yrs. = 12%								
Wyckerpoort	4.015	0–24 yrs. = 31%	14%	51%	49%	9%	24%	36%	12%	43%
		25–64 yrs. = 49%								
		≥65 yrs. = 20%								
Wittevrouwenveld	5.706	0–24 yrs. = 36%	19%	56%	29%	15%	24%	53%	18%	57%
		25-64 yrs. = 53%								
		≥65 yrs. = 11%								
Comparison group										
Mariaberg	4.965	0-24 yrs. = 33%	14%	58%	28%	17%	37%	57%	17%	51%
		25-64 yrs. = 49%								
		≥65 yrs. = 17%								
Pottenberg	2.415	0-24 yrs. = 24%	18%	58%	21%	18%	37%	53%	28%	56%
		25-64 yrs. = 53%								
		≥65 yrs. = 22%								
Caberg	3.410	0-24 yrs. = 33%	20%	60%	23%	22%	40%	58%	16%	58%
		25-64 yrs. = 51%								
		≥65 yrs. = 17%								
Malberg	5.345	0-24 yrs. = 23%	13%	55%	16%	19%	40%	51%	21%	58%
		25-64 yrs. = 50%								
		≥65 yrs. = 28%								
Maastricht and the Netherlands										
Maastricht	122.533	0-24 yrs. = 30%	10%	14.3%	40%	12%	21%	45%	14%	46%
		25-64 yrs. = 49%								
		≥65 yrs. = 20%								
Netherlands	17.205.411	0-20 yrs. = 23.5%	12%	9%	34%	10%	21%	44%	10%	49%
		21-64 yrs. = 59%								
		≥65 yrs. = 17.6%								

(Source: CBS – Statistics Netherlands, 2016; National Monitor Public Health Services South Limburg, 2017; Municipality Monitor Social Domain Maastricht, 2018)

^a^*Threshold low income = 1030 euro’s for a one person household and 1930 for a couple with two children* [31]

^b^*Being overweight: a Body Mass Index between 25 and 30* [41]

### Measures and data collection

#### Population health and (experienced) quality of care

Effects of the Blue Care ICA on population health and experienced quality of care are being measured. The primary outcome measure is health-related quality of life, measured by the 12-item Short Form Health Survey version 2 (SF-12v2) [33]. The SF-12v2 is a multidimensional generic measure of health-related quality of life containing 12 items derived from the SF-36v2. Two summary scores can be generated, i.e., the Physical Component Summary Scale Score and Mental Component Summary Scale Score. Secondary outcome measures are the 5-level EQ-5D version (EQ-5D-5 L) [34], Resilience Scale (RS-scale) [35], perceived quality of care [36] and the Spiderweb diagram of Positive Health [25, 27]. Outcomes are assessed at baseline (T0), after 12 months (T1), 24 months (T2) and after 38 months (T3) in the intervention and comparison neighbourhoods using both digital and paper questionnaires. Table 2 shows a complete overview of the measurement tools and dimensions of the effect study.

**Table 2:** Outcome measures effect measurement

Dimension	Measure	Items/subscales	Timing data collection^a^
Demographic and background Characteristics	Demographic and background Characteristics	Gender, date of birth, household composition, education, participation, country of birth, medical care avoidance, 10 – point scale rating satisfaction of neighbourhood	T0, T1, T2, T3
Primary outcome measure effects			
Population health	SF-12v2	12 items, physical and mental component	T0, T1, T2, T3
Secondary outcome measure effects			
Population health	EQ-5D-5 L	6 items, mobility, self-care, usual activity, pain/discomfort, anxiety/depression. VAS-scale rating perceived health	T0, T1, T2, T3
Population health	Resilience Scale (RS-scale)	25 items personal competence, acceptance of self and life	T0, T1, T2, T3
Population health	Spiderweb instrument of Positive Health	6 items bodily functions, mental functions/perception, existential being, quality of life, participation, daily functioning	T0, T1, T2, T3
Experience of care	Quality of care	10 point scale, grading the quality of care, trust in caregivers	T0, T1, T2, T3

^a^T0 (baseline), T1 (after 1 year), T2 (after 2 years), T3 (after 3 years)

#### Costs

To investigate the influence of the Blue Care ICA on per capita costs, two levels for computing reimbursements retrospectively are distinguished: population level and user level. First, the amount and the trend in the annual reimbursements during the intervention period (T0-T3) in the health and social care domain (care and support financed under the Health Insurance Act by the health insurance company and the Social Support Act/Youth Act/Participation Act/Public Health Act by municipalities) are computed on a population level for the citizens in the four neighbourhoods where the Blue Care ICA is being implemented (total amount as well as mean per citizen). These reimbursements will be compared to the reimbursements in the reference period 2015–2016 and to the comparison neighbourhoods (risk –adjusted standardized reimbursements) as well as the national figures to investigate whether a break in the trend of increasing costs will appear at the population level. Furthermore, the annual mean volumes of used medical services and services in the social care domain will be computed for both the intervention and comparison neighbourhoods to investigate whether changes in the number of used services took place (user level).

Health care costs with data related to health and social care, as well as reimbursement data will be collected via existing databases and registration systems from the health insurer VGZ, the municipality of Maastricht, and Statistics Netherlands (CBS, national statistics database with an overview of volumes and costs of both health as social care).

#### Work satisfaction of professionals

Work satisfaction of the professionals in the neighbourhoods and the facilitators and barriers for the implementation of Positive Health within daily working routines will be investigated. Both overall work satisfaction and work satisfaction during the specific sub-projects will be investigated. Data will be collected using semi-structured interviews with health and social care professionals in the neighbourhoods. A wide range of social care professionals (e.g.: general practitioner, nurse, social care worker) will be interviewed to get a broad understanding of the facilitators and barriers.

### Sample size considerations effect measure

The sample size calculation for the study is based on the primary outcome measure health-related quality of life, measured with the SF-12v2. To demonstrate a clinically relevant treatment difference of 3.0 on the Mental Component Summary Scale Score, given SD = 8.88 [36], and an alpha of 0.05 and a power of 0.80, the minimally required number of participants is 138 per study. In order to demonstrate the effects of the ICA on the subpopulation level with respect to age (18–65 years vs. 65 years and older), 138 participants per age category are required, resulting in a total of 2 × 138 = 276 participants per study group. The total required sample size (intervention + comparison group) is therefore 552 (2 × 276).

**Fig. 4:**
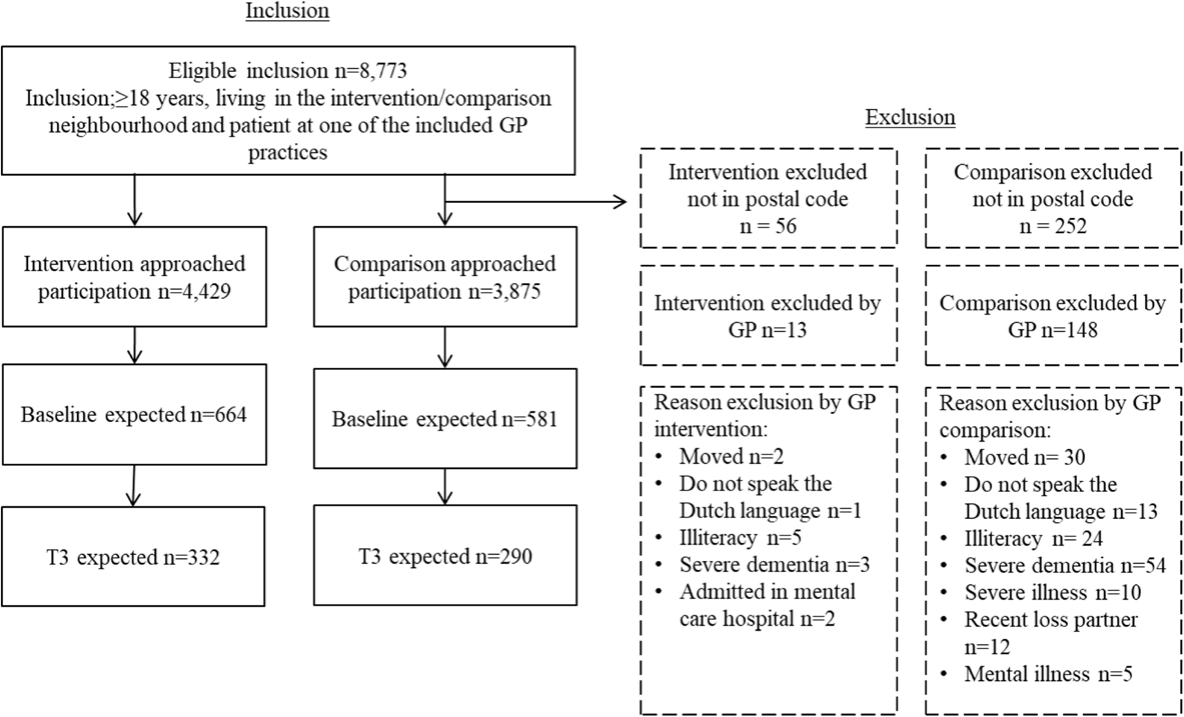
Flow chart of the study

It is expected that 15% of all citizens approached for participation by means of an information letter and consent form will agree to participate. Additionally, a drop-out rate of 50% between T0 and T3 is expected. Accounting for drop-out, 1104 citizens need to be enrolled at T0 (552 per study group). Based on the expected response rate of 15% across all age groups and neighbourhoods, 7360 citizens (3680 per study group) need to be approached for participation. As the required number of participants to be approached per study group closely resembled the number of clients in the general practice population that were eligible for inclusion (at least for the comparison group), we decided to approach all eligible citizens to prevent selection bias. Figure 4 presents a flow chart of the study based on the actual number of citizens approached for participation, the expected response rate and the expected drop-out rate.

### Process evaluation

The second part of this research evaluates the process of implementation of the Blue Care ICA in the intervention neighbourhoods on macro (e.g. policymakers), meso (e.g. managers and directors) and micro (e.g. professionals and citizens) level. For understanding this process we use various methods of data collection, such as participant observation, analyzing meeting notes, semi-structured interviews, focus groups, and a citizen panel. We aim to capture the context in which Blue Care ICA will unfold and the thinking and practices that are tied to it. This allows for a more thorough understanding of both intervention delivery as well as aspects of the context in which the intervention is embedded. The Consolidated Framework for Implementation Research (CFIR) is used as an overarching framework to formulate a process evaluation plan. The CFIR model consists out of five major domains: intervention characteristics, outer setting, inner setting, characteristics of the individuals involved and the process of implementation [29, 37, 38]. These five domains comprise a total of 39 sub-domains. Table 3 shows an overview of the process evaluation questions, accompanying methods and timing of data collection. To conclude, the CFIR model provides a pragmatic structure for the complex, interacting, multi-level ICA in a ‘real world setting’ and works as an overarching, additional model for validated and frequently used implementation theories in public health, such as the innovation theory of Rogers [39] and the Re-Aim model [40]. Moreover, the CFIR model can be used for both summative and formative purposes, which is a major advantage for this research since sub-projects are developed over time and formative evaluation allows the researcher to give intermediate feedback to the implementers of the ICA. This contributes to the valorisation of this study.

**Table 3:** Framework Process evaluation

Topic	Questions	Method/Source	Timing data collection
• Characteristics of the projects in the Blue Care ICA	- What are the characteristics of the projects developed ‘on the go’ in terms of the targeted audience and topic? And how do they contribute to the Quadruple Aim?	Observations, existing data sets, and semi-structured interviews with policymakers, health and social care professionals, citizens	T0, T1, T2, T3 (ongoing between 2017 and 2020)
• Citizen needs of the people living in the intervention neighbourhoods, • The influence of external policies on the Blue Care ICA	- Does the ICA meet the needs of the citizens and professionals living and working in the neighbourhoods? - Are there other contextual or environmental features which play a role in the experienced quality of life of citizens living in the neighborhood? - How do external strategies (policy and regulations / government) influence the pilot and the implementation?	Citizens: Citizen panel *n* = 10 × 2 (1 panel per two neighbourhoods) Policy: observations during existing board and financial sponsor meetings	Citizen Panel: T1, T2, T3 (meet 2 times a year) Policy: T0, T1, T2, T3 (ongoing between 2017 and 2020)
• Governance structure of the Blue Care ICA	- How is the governance model structured, how does this develop in these four years and what are the different roles in this structure (leadership, champions etc.)?	Observations during existing board and financial sponsor meetings, semi-structured interviews	T0, T1, T2, T3 (ongoing between 2017 and 2020)
• Implementation of the bottom up approach, • Collaboration, interaction and communication on macro, meso and micro level, • Use and implementation of Positive Health by health and social care professionals	- How are citizens and professionals actively involved and attracted in the pilot process (goal is bottom – up approach)? - How do healthcare and social service providers collaborate in the social domain at macro, meso and micro level and to what extent are they able to prioritize mutual interests above their own organisational interests? - How do professionals deliver person-centred care and support from a generalist perspective according to the principles of Positive Health, how do they implement Positive Health into their daily routine, and what is the influence on the type of care and support provided to citizens?	Participant observations at all levels, analyzing meeting notes, semi-structured interviews, focus groups with health and social care professionals, citizen panel	T0, T1, T2, T3 (ongoing between 2017 and 2020)

### Data analysis

Descriptive statistics will be computed to describe the characteristics of the target population in participating neighbourhoods (e.g., age, socioeconomic status, ethnicity, household composition) and to compare the intervention and comparison neighbourhoods at baseline. Relevant statistical tests (e.g., t-test, chi-square, analysis of variance, regression analysis) will be applied to analyse effects on the primary and secondary outcome measures (level of significance is 0.05; two-tailed). Data will be analysed according to the intention-to-treat principle. This means that all subjects included in the intervention group will be analysed as intervention subjects irrespective of whether they received the intervention or not. This allows for judging whether the neighbourhood population as a whole benefits from the Blue Care ICA, irrespective of whether citizens actually received (elements of) the intervention.

In all analyses, possible baseline differences between citizens from intervention and comparison neighbourhoods will be corrected for. In addition, subgroup analyses will be performed to investigate whether certain groups of citizens benefit more from the integrated community approach than others (i.e., according to age, gender, type of problems, socioeconomic status). Qualitative data will be analysed using narrative descriptions of procedures and meetings, identifying themes in the focus group with professionals, citizens’ panels, and semi-structured interviews with citizens, professionals, managers, and directors. Interviews will be transcribed verbatim and Nvivo 12.0 will be used for the analysis.

Descriptive statistics will be used to describe the total amount of reimbursement at the population level over time as well as (changes in) the proportion of reimbursement in the social domain (on account of the Social Support Act, the Youth Act and the Participation Act) and in the health care domain (on account of the Health Insurance Act, e.g., primary care, hospital care, mental health care, medication and diagnostics).

### Registration, ethics and data management

The study is registered with the Dutch Clinical Trial Registry (NTR) (https://www.trialregister.nl/trial/6359 NTR6543; registration date July 25th, 2017).The current study protocol (version 1) is the version for which The Medical Ethical Committee (METC) of the Maastricht University Medical Center (MUMC+) waived the need for ethics approval. Data will be collected between 2017 and 2020. Any amendments to the original protocol will be announced to the METC and the NTR. The research team located at the Department of Health Services Research at Maastricht University will be responsible for the data management and this will be in accordance with the data – management protocol of Maastricht University. Confidential data is stored in a secure place, separated from personal response datasets. The main researcher and a trained research assistant will be the only persons who have access to the personal confidential data. The primary funder of this study is The Netherlands Organisation for Health Research and Development (ZonMw). ZonMw has no role in the intervention, research nor the publication of the data. Data retention and possible dropouts will be minimised by a postal reminder when there is no initial response from the participants. Collected and analysed data on a group level, which is not traceable to individuals, will be shared in a ‘main outcome report’ with the citizens, professionals and other stakeholders of the Blue Care ICA.

## Discussion

In this paper, the design of a mixed-methods study, including a prospective, quasi-experimental study and a thorough process evaluation, is presented. It aims to measure the effects of the Blue Care ICA on Quadruple Aim outcomes and to investigate the implementation process of Blue Care ICA in four low socioeconomic neighbourhoods of Maastricht.

The Blue Care ICA, as described here, poses challenges for the design of an evaluation study to investigate effects and the implementation process. First, exposure to the Blue Care ICA at the individual level cannot be manipulated (i.e., randomised) by a researcher, as it would restrict obtaining an objective and real-life picture of the distribution of budgets, delivery of care or services in the health and social care domain, and the development of initiatives by citizens. Experimental manipulation at the neighbourhood level is also not feasible, as these neighbourhoods and the organizations involved have signed an intention to implement the Blue Care ICA, based on the ideology of Positive Health and by combining budgets. Furthermore, instead of a fixed intervention with clear guidelines for implementation, variability in content and implementation can be expected, as the projects of the Blue Care ICA need to evolve over time and require translation into daily working routines during the process of implementation. Additionally, implementation of the intervention can be challenged by external and internal factors (e.g., political elections or re-organisations of the organizations important stakeholders participate in). Correspondingly, we do not know beforehand which specific sub-projects will be developed, since the Blue Care ICA is a bottom-up approach, trying to fill in the gaps where the existing social and healthcare system fails in these four neighbourhoods. The projects are developed to address the Quadruple Aim goals and are also created based on the needs of citizens and professionals instead of implementing a fixed theory or intervention protocol. This makes it challenging to determine beforehand which activities can be an object of research. Nevertheless, the chosen research design presented in this article provide a thorough research direction and guidance that also allows enough room for the researcher to incorporate the research needs of the sub-projects which are developed ‘on the go’. The process evaluation will provide insight into the extent to which these factors contributed to the outcome and effects of the intervention. Results of the effect and process evaluation will become available in 2020.

## Abbreviations

AHC: Accountable Health Community
CBS: Statistics Netherlands
CFIR: Consolidated Framework for Implementation Research
CMS: Centers for Medicare and Medicaid Services
EPD: Electronic Patient Systems
EQ-5D-5 L: The EuroQol- Five Dimensions- Five Levels
ICA: Integrated Community Approach
IHC: Integrated Health Care
METC: The Medical Ethical Committee
MUMC+: Maastricht University Medical Center
NTR: The Dutch Clinical Trial Registry
PHS: Public Health Services
RS: Resilience
SD: Standard Deviation
SDG: Sustainable Development Goals
SES: Socioeconomic Status
SF-12v2: 12-item Short Form Health Survey version 2
UN: United Nations
WHO: World Health Organization
ZonMw: The Netherlands Organisation for Health Research and Development
